# Volumetric Analysis of the Jaws in Skeletal Class I and III Patients with Different Facial Divergence Using CBCT Imaging

**DOI:** 10.1155/2022/2416555

**Published:** 2022-05-28

**Authors:** Rama Yasser Alhawasli, Mowaffak A Ajaj, Mohammad Younis Hajeer, Assil Mohammad Raef Al-Zahabi, Luai Mahaini

**Affiliations:** Department of Orthodontics, University of Damascus Dental School, Damascus, Syria

## Abstract

**Aim:**

The main objective was to evaluate any possible maxillary or mandibular volumetric difference between hyperdivergent skeletal Class III (CIII), normodivergent skeletal CIII, hypodivergent skeletal CIII, and normodivergent skeletal Class I (CI) patients using cone-beam computed tomography (CBCT) images. Also, the secondary objective was to investigate any possible correlation between CBCT-derived lateral cephalometric variables and the mandibular and maxillary volumes (MdV and MxV, respectively).

**Materials and Methods:**

80 CBCT images of patients between 18 and 32 years of age were taken with one CBCT imaging device (Scanora 3D®, Soredex, Tuusula, Finland). The sample consisted of four groups: 20 hypodivergent skeletal CIII (11 males and 9 females), 20 normodivergent skeletal CIII (7 males and 13 females), 20 hyperdivergent skeletal CIII (8 males and 12 females), and 20 normodivergent skeletal CI (5 males and 15 females). The volumes of both jaws and the ratio of MxV/MdV were obtained using Mimics™ 19 software (Materialise, NV, Belgium), and 2D variables were obtained from CBCT-derived lateral cephalogram using AudaxCeph™ software (Orthodontic software suite, Ljubljana, Slovenia). One-way ANOVA test and Kruskal–Wallis analysis were employed to detect any possible significant difference between the volumetric variables, whereas Pearson's and Spearman's correlation coefficients were calculated to detect any possible relationship between the 2D variables and the volumetric measurements.

**Results:**

There were no statistically significant differences in the maxillary volume or maxillary/mandibular ratio between the four groups (*p*=0.081 and 0.432, respectively). There was a significant difference in MdV between CIII hypodivergent (higher mean) and CIII hyperdivergent (*p*=0.039). There were some correlations between the MdV and 2D variables in the four studied groups especially in the posterior facial height (S-Go) and the facial depth (N-Go). There were some weaker correlations between the MxV and some 2D variables in the CIII hypodivergent and hyperdivergent groups.

**Conclusions:**

The mandibular volume of the Class III hypodivergent patient was significantly greater than that of the Class III hyperdivergent patients. Correlations between the maxillary or mandibular volumes were found with some of the 2D variables. The volume of both jaws increased when the maxillofacial complex moved toward a horizontal growth pattern.

## 1. Introduction

There are many characteristics that distinguish a Class III malocclusion (CIII) from other craniofacial problems [1]. A correct patient assessment is essential in these cases to make the right diagnosis and reach the perfect treatment plan that leads to the optimum functional and esthetic results [2, 3].

Researchers attributed the etiology of the Class III malocclusion to morphologic or positional disharmony between the maxilla and the mandible during the growth period [4]. Since there are many variations in Class III morphology, it became a necessity to use the wide applications of 3D imaging in orthodontics to study the volumes of the jaws for a better understanding of the problem [5–8] that may lead to more accurate treatment techniques [9].

Research works have tried to connect cranial morphology with different types of malocclusions, and some have studied the relationship between Class III malocclusion and the cranial base measures [10–13]. However, all of these studies have used linear measurements taken from two dimensional radiographies, as conventional cephalogram was the primary source to determine the morphological characteristics of Class III malocclusion [14]. This type of radiography offered no quantitative volumetric information for the maxilla or mandible. Later on, cone-beam computed tomography (CBCT) imaging systems were developed to be used as a diagnostic and analytical tool for the craniofacial complex [15–17]. This radiographic technique offered the chance to gain volumetric measurements that can connect the jaw morphology with malocclusion.

Few pilot studies have used CBCT imaging to evaluate maxillary and mandibular volumes in different types of sagittal malocclusions. However, the samples in these studies were relatively small [6, 18]. Other researchers that studied the mandibular volume in different sagittal malocclusions have not considered the impact of the vertical skeletal pattern on the volume [19, 20]. When the maxillary volume was taken into account, it was calculated by segmenting the upper jaw anterio-posteriorly without considering the complex anatomy of the maxilla [6, 18] except for one study by Nahas et al. that included the different processes of the maxilla in the volume [21]. However, Nahas et al.'s study was conducted on Class II hyperdivergent patients in comparison with Class I (CI) and did not evaluate Class III malocclusion patients in their sample.

Therefore, the primary aim of this work was to evaluate any possible maxillary or mandibular volumetric difference between hyperdivergent skeletal CIII, normodivergent skeletal CIII, hypodivergent skeletal CIII, and normodivergent Class I patients using CBCT images. Whereas the secondary aim was to investigate any possible correlation between CBCT-derived lateral cephalometric variables and the mandibular and maxillary volumes.

## 2. Materials and Methods

### 2.1. Study Design and Setting

This study was an observational cross-sectional study for analytical and descriptive purposes. It was conducted in Syria at the Orthodontic Department in Damascus University, Faculty of Dentistry, after obtaining the necessary approval from the Local Research Ethics Committee at the University of Damascus Dental School, Syria (Ref no. UDDS513-09072017/SRC-3762). This research was funded by the University of Damascus Dental School Postgraduate Research Budget (Ref no. 84130694351DEN).

### 2.2. Sample Size Calculation

The sample size was calculated using Minitab V16 Inc. (State College, Pennsylvania, USA). The lowest volumetric difference of the mandible that should be detected is 10000 mm^3^. Since the variance of this variable in a previous study was approximately 9000 mm^3^ [20] and by using the hypotheses*p* value <0.05, the power of study is 80% and the One-way ANOVA test was conducted between the four groups. For those assumptions, it was found that the required sample size for each group is 19 patients and this number was rounded to 20 patients in case one 3D image was deemed unsuitable for volumetric assessment.

### 2.3. Collection of CBCT Images

CBCT images were collected in two steps. The first step included gathering all the CBCT images of patients who had been referred to the Orthodontic Department at the Faculty of Dentistry (Damascus University, Damascus, Syria) between the years of 2009 and 2019. All the patients were between 18 and 32 years of age. Those CBCT images were taken for orthodontics or orthognathic surgery purposes. All of these images were taken using the same CBCT imaging system (Scanora 3D, Soredex, Tuusula, Finland) with 15 mA and 85 kV, 40 seconds of exposure time, and isotropic voxel size of 0.25 × 0.25 × 0.25 mm.

The images that were gathered were 870 in total. All images that have jaw fractures, cleft lips and/or palates, or extended cystic lesions in the mandible or maxilla were eliminated. In addition, images of patients who have congenital disorders, systemic diseases, and history of previous orthodontic treatment or missing teeth were also discarded. A quick assessment of the sagittal skeletal relationship was accomplished using the ANB angle by locating the three landmarks (A, B, and N) in order to exclude skeletal Class II patients.

By the end of the first step, 340 images were found preliminarily suitable. The second step included deriving cephalometric images from the CBCT images and running detailed analysis to acquire the exact sampling frame. For the patients to be accepted in the Class I group had the following cephalometric characteristics: ANB = 2–4°, Y-axis angle = 60–70°, Bjork sum = 390–400°, and MM (mandibular-maxillary angle) = 22–30°. However, the CIII patients had an ANB ≤ 1.5°. The hypodivergent group met at least two of the following three cephalometric characteristics: Y-axis angle <60°, Bjork sum <390°, and MM < 22°. The normodivergent group met at least two of the following: Y-axis angle = 60–70°, Bjork sum = 390–400°, and MM = 22–30°. The hyperdivergent group met at least two of the following: Y-axis angle > 70°, Bjork sum > 400°, and MM > 30°. The cases in between were not accepted in this study.

Later, a sampling frame of 155 suitable CBCT images was achieved (including 34 CBCT images of patients with normodivergent skeletal Class I malocclusion and 121 CBCT images of patients with Class III malocclusion encompassing the three different types of maxilla-mandibular divergence). Then, a disproportionate stratified random sampling was employed to create the following four groups: the first group included 20 CBCT images of patients with hypodivergent skeletal Class III malocclusion; the second group consisted of 20 CBCT images of patients with normodivergent skeletal Class III malocclusion; the third group had 20 CBCT images of hyperdivergent skeletal Class III malocclusion patients; and the last group included 20 CBCT images of normodivergent skeletal Class I patients.

### 2.4. CBCT-Derived Lateral Cephalograms and the 2D Analysis

**Figure 1:**
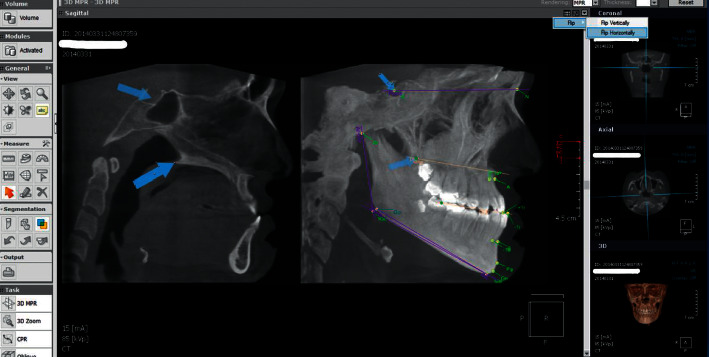
Left image: Two arrows were used to point out the exact places of points S (Sella) and PNS (Posterior Nasal Spine) and these arrows were preserved during slice manipulation. Right image: The thickness of the sagittal view was increased to 100 mm to get a CBCT-derived cephalometric image while keeping the arrows stable in their places in order to help in identifying points S and PNS accurately.

Files were saved in Digital Imaging and Communications in Medicine (DICOM) format, and the lateral cephalogram was derived from the CBCT image by OnDemand 3D® program (CyberMed, Seoul, Korea). The image was redirected so that the midsagittal plane passes through the anterior nasal spine (ANS) and the posterior nasal spine (PNS) in the Maximum Intensity Projection (MIP). This orientation of CBCT images was done according to the method proposed by Echevarria-Sanchez et al. [22]; then, the image was flipped horizontally (so that the patient's face was looking to the right) and on the “sagittal view” (which actually represented the midsagittal plane), “arrows” were used to point out in the exact places of points S (Sella) and PNS (Posterior Nasal Spine) and these arrows were preserved during slice manipulation. Next, the thickness of the sagittal view was increased to 100 mm to get a CBCT-derived cephalometric image while keeping the arrows stable in their places representing the accurate positions of the points S and PNS (Figure 1).

**Figure 2:**
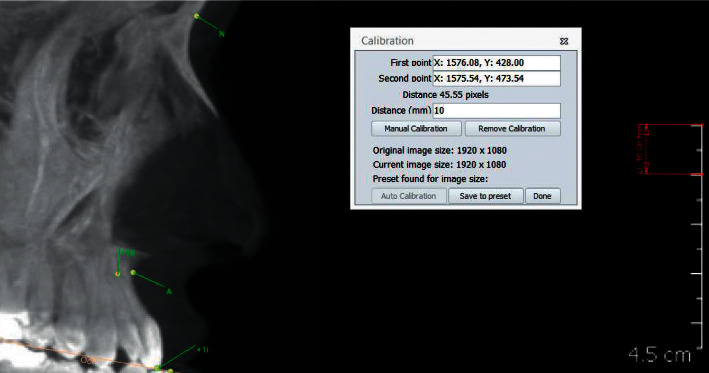
Calibrating the cephalometric image using AudaxCeph™.

The CBCT-derived cephalograms were imported into Audaxceph® V3.4.2.2710 (Orthodontic software suite, Ljubljana, Slovenia) for tracing. The magnification ratio was determined by using the ruler on the side of the image and inserting the right value in the calibration section (Figure 2). All the cephalometric analysis was conducted by one researcher (R.Y.A.). Eleven linear and angular cephalometric measurements were performed. The definitions of these measurements were taken from the work of Jacobson and Jacobson [23] and Riolo et al. [24] and are shown in Figure 1 and explained in Table 1.

### 2.5. 3D Volumetric Analysis from CBCT Data

**Figure 3:**
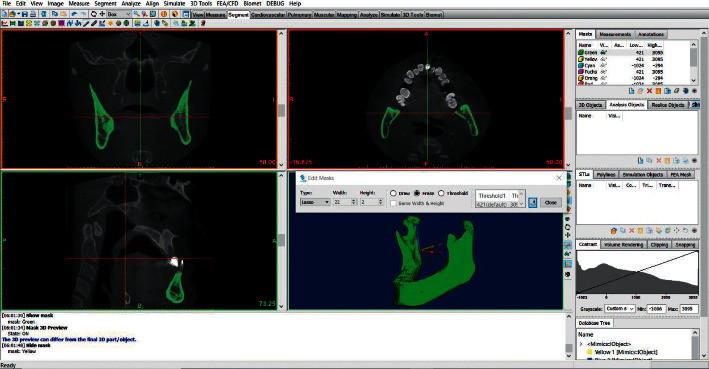
The mandibular bone after segmentation using edit mask tool.

**Figure 4:**
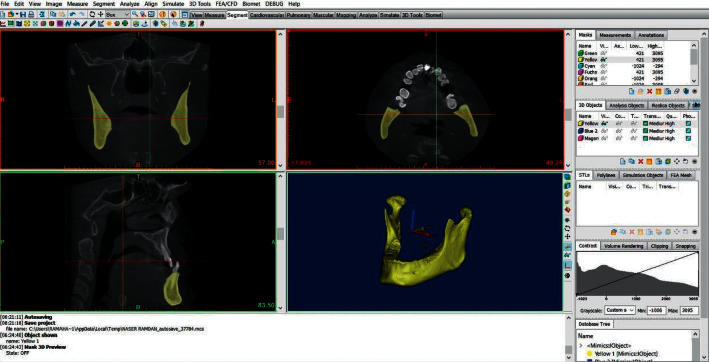
The solid final mandibular bone.

**Figure 5:**
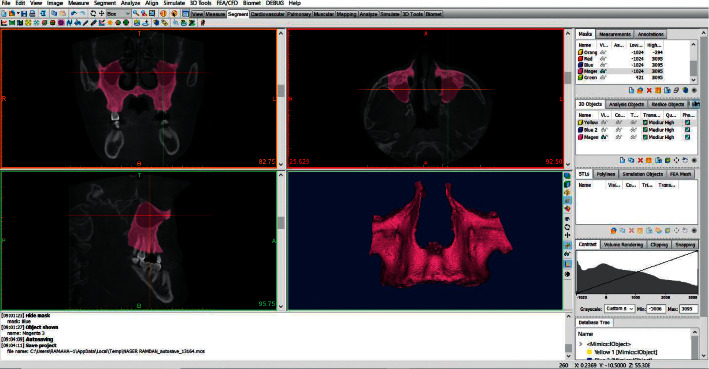
The solid final maxillary bone.

**Figure 6:**
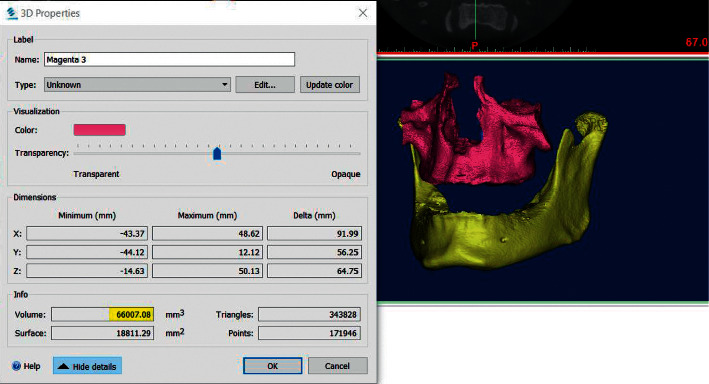
The volume of the maxillary bone shown in the 3D properties box (highlighted in yellow). In addition, the 3D objects are shown in the window for both the mandible and the maxilla.

In order to calculate the Maxillary Volume (MxV) and the Mandibular Volume (MdV), the DICOM files were imported into Mimics™ 19 program (Materialise, NV, Belgium). The maxillary bone boundaries were located at its anatomical sutures with the neighboring bones, similar to the method proposed by Nahas et al. [21]. As a next step, the whole upper jaw was measured (including the maxilla and palatal bones); the maxillary sinus was included without the inclusion of teeth crowns. The mandibular volume was measured with the inclusion of the condyles and without the teeth crowns. A semimanual technique was used to segment the target bone from the surroundings. First, a single threshold value was selected based on a local gray level value and image gradient to create a mask of the desired bone. Later, the surroundings of the bone were erased by using Edit Mask-Erase-Lasso Tool in the 3D window to get a mask that contains the outline borders of the bone (Figure 3). Then, Sang et al.'s technique was used in drawing or erasing the mask manually layer by layer on at least two orientations to get a solid and complete entity of the bone with all of the internal cavities filled [25]. The technique of Sang et al. has been already validated (Figures 4 and 5). The volumetric measurement was carried through the Mimics™ automatic function after building a 3D object of the bone (Figure 6).

### 2.6. Statistical Analysis

Shapiro–Wilk normality tests were first conducted. The one-way ANOVA test was employed to detect any possible significant difference in the MxV and the ratio of the MxV/MdV between the four studied groups, whereas Kruskal–Wallis tests were carried out to detect any significant difference in the MdV between the four groups due to the inability to run one-way ANOVA tests. When significant differences were detected, Dann Test with Bonferroni correction was conducted to determine which groups have a significant difference between. Pearson's correlation coefficients (or the nonparametric equivalent; Spearman's correlation coefficients) were calculated to detect any possible relationship between the 2D variables and the volumetric measurements.

### 2.7. Reliability of the Method of Volume Calculation and the 2D Measurements

To investigate the reliability of the employed volume calculation procedure, the MxV and the MdV of 10 randomly selected patients were measured twice with a time interval of six weeks by the same principal researcher (R. Y. A.). In addition, the 2D measurements of the CBCT-derived cephalograms were also remeasured after six weeks by the principal researcher (R. Y. A.) for 10 randomly selected patients. Intraclass correlation coefficients (ICCs) were used to determine the intraobserver reliability, whereas the paired *t*-test was employed to detect any significant difference between the two assessment times (i.e., systematic error).

## 3. Results

Each of the four studied groups included 20 CBCT images. The first group of patients with hypodivergent skeletal Class III malocclusion had 11 males and 9 females with the mean age being 24.95; the second group of patients with normodivergent skeletal Class III malocclusion included 7 males and 13 females with the mean age being 24.46; the third group of hyperdivergent skeletal Class III malocclusion patients consisted of 8 males and 12 females and the mean age was 22.82; and the last group of normodivergent skeletal Class I patients had 5 males and 15 females with the mean age of 24.27.

Regarding the assessment of method reliability, there were no significant differences between the two assessment times for the MxV or MdV (i.e., no systematic error; *p* > 0.05). Also, no significant differences were found between the two assessment times regarding the 2D cephalometric measurements (*p* > 0.05). The reliability analysis confirmed an excellent agreement between the two readings (ICC ranged between 0.997 and 0.999; Table 2).

Descriptive statistics of the MxV and MdV as well as maxillary/mandibular volumetric ratios are shown in Table 3. There were no statistical significant differences in the maxillary volume or maxillary/mandibular ratios between the four groups. But there was a statistically significant difference in mandibular volume between CIII hypodivergent and CIII hyperdivergent (*p*=0.039) with the mean MdV being greater in the CIII hypodivergent group (Table 4).

The highest number of correlation between the 2D variables and the volumetric measurements was found in the CIII Hypo-divergent group with the strongest ones between the mandibular volume and the anterior and posterior facial lengths and facial depth in addition to negative strong correlations between the MdV and the inclination of the maxilla, rotation of the mandible, and the sum of Bjork. As for the CIII Normo-divergent group, the strongest correlations were between the both jaws' volumes and the anterior and posterior facial lengths and the facial depth (Table 5).

As for the remaining groups, the strongest correlations were only between the mandibular volume and the posterior facial length and the facial depth (Table 6).

## 4. Discussion

To the best of our knowledge, this is the first study to calculate the full complicated anatomical volume of the upper jaw, including the maxillary bone, palatal bone, and the maxillary sinus. The earlier studies of Nair and Deguchi calculated the maxillary bone volume by sectioning it anterio-posteriorly and including the lower part of the maxilla only [6, 18]. Nahas et al. conducted the first study to measure the maxillary volume including the anatomical processes. However, the intraosseous sinuses were not filled [21]. Not including these sinuses in the volumetric calculations left their measurements apparently less than the actual size.

Therefore, the current study aimed to acquire a solid volume by filling all the spaces in the bone slice by slice in the axial view as this has been shown to be a validated method in giving real-life volumes [25]. The maxillary sinus was intended to be included in the MxV as it is anatomically considered an air space inside the maxillary bone and is thus a part of it [26]. In addition, the posterior borders that surrounded the maxillary sinuses were found to be too thin in the CBCT images to the extent that excluding these sinuses would distort the calculation of the bony segments at these areas.

As for the lower jaw, the technique that was used to get the MdV assured that all osseous hollows in the mandible were completely filled to get true volumes [25]. Also, condylar volumes were considered part of the MdV as was followed in previous studies. Teeth crowns were cut out at the level of the alveolar bone crest to prevent artifacts and distortions related to the presence of metallic restorations and crowns [6, 18–21].

There were a statistical significant difference in the MdV between CIII hyperdivergent patients and CIII hypodivergent patients (*p*=0.039). This result was similar to a recent study [20] which found a difference in MdV between different vertical patterns. Some other factors that can differ between vertical and horizontal growth patterns include the masseter volume, type of muscle fibers [27], and occlusion force [28]. Research studies showed an inverse relationship between vertical growth pattern and the length of the masseter muscle [29, 30]. Hyperdivergent patients have weaker muscle activity [18], and this leads to overeruption of the posterior teeth and less apposition of periosteal bone in the angular region of the lower jaw [31]. On the contrary, hypodivergent growth patterns have stronger masseter muscles which lead to thickness of the alveolar ridge and cortical bone [32]. This all could explain the observed difference of MdV in the current study.

There was no statistical significant difference in maxillary volume between the four studied groups. Although the segmentation method differs from others' studies [6, 18], the current finding agrees with the previous pilot studies. This result can be explained that CIII patients have positional, but not volumetric, problem in the maxillary complex. Thus, it is always helpful to think of treatment plans that change the position of the maxilla (e.g., facemask [33]) not the size of it.

There was no statistical significant difference in maxillary/mandibular volume ratio among the studied groups. This result differs from previous pilot studies [6, 18]; this can be attributed to the small sample size of these pilot studies or the difference in the way of calculating the maxillary volume.

### 4.1. Correlation between the Maxillary and Mandibular Volumes and the 2D Analysis

In the Class I normodivergent group, there was a strong positive correlation between the MdV and the posterior facial height (S-Go) and facial depth (N-Go) and negative correlation between the MdV and the gonial angle and Bjork sum. These correlations indicate that the MdV increases when there is a tendency toward horizontal growth pattern. Previous studies showed negative correlation between masseter force and both gonial angle [34] and Bjork sum [35] which is indirectly similar to the current findings.

In the Class III normodivergent group, there was a strong positive correlation between MdV, MxV, and anterior facial height (N-Me), posterior facial height (S-Go), and facial depth (N-Go). Previous studies showed positive correlation between both posterior facial height and facial height index [34]. Therefore, every increase in posterior facial height can lead to bony deposition (probably due to the increase in masseter muscle strength) and this can explain the increase in MxV and MdV. And as described previously, studies have shown an inverse relation between vertical growth pattern and masseter length [29].

In the Class III hypodivergent group, there was a strong negative correlation between MdV and (Md-SN), (Mx-SN), and Bjork sum and a positive correlation between MdV and anterior facial height (N-Me), posterior facial height (S-Go), and facial depth (N-Go). This indicates an increase in the MdV as the growth pattern decreases as described above. Also, there was a strong negative correlation between the MxV and (Mx-SN) which goes in line with what was described earlier about the decrease in the MxV with the hyperdivergent growth pattern. When evaluating the results of the Class III hyperdivergent group, there was a strong positive correlation between MdV and anterior facial height (N-Me), posterior facial height (S-Go), and facial depth (N-Go), which reflected the same trends that were described above.

### 4.2. Limitations

The possible difference between males and females in the volumetric assessment was not evaluated in the current study. Therefore, it is recommended for future research work to have a larger sample size to allow for a gender-based analysis. In addition, the skeletal Class I patients included in this study had malocclusions that required the use of CBCT images for diagnostic purposes rather than depending on CBCT images of healthy Class I individuals, which is ethically unacceptable. In addition, this research work was only confined to evaluating skeletal volumes in CIII patients with different levels of divergence in comparison with the CI normodivergence patients. Additional studies are required to evaluate jaw volumes in other skeletal classes of malocclusion in the sagittal plane in conjunction with different patterns of vertical growth.

### 4.3. Generalizability

The generalizability of this study may be limited due to the fact that the CBCT images were all recruited from one teaching hospital with one ethnicity being studied. In addition, all images were taken using one CBCT apparatus with one researcher performing model segmentation and data analysis.

## 5. Conclusions

1. Class III patients with hypodivergent facial pattern had greater mandibular volume than those with hyperdivergent pattern
2. There were no statistical differences in the maxillary volume or the MxV/MdV ratio between the four evaluated malocclusion groups
3. The strong correlations between the mandibular volume and the posterior facial length and facial depth in all four studied groups indicate that the mandibular volume increases when the maxillofacial complex moves toward horizontal growth pattern
4. The strong correlations between the maxillary volume and the posterior facial length and facial depth in the CIII normodivergent group suggest that the maxillary volume also increases when the maxillofacial complex moves toward horizontal growth pattern

## Figures and Tables

**Table 1:** The linear and angular measurements measured on the CBCT-derived cephalometric images^*∗*^.

SNA	The sagittal position of the maxilla in relation to the base of the skull
SNB	The sagittal position of the mandible in relation to the base of the skull
ANB	The difference between the above two angles, it represents the sagittal relationship between the jaws
Y-axis	The angle that is formed from the points (N-S-Gn) according to Jarabak
MM angle	The angle between the maxillary and mandibular planes
Md.SN	(S-N : Go-Me) The angle between the mandibular plane and the anterior basal plane
Mx.SN	(S-N : Spp) The angle between the maxillary plane and the anterior basal plane
N-Me	The anterior facial height (mm)
S-Go	The posterior facial height (mm)
N-Go	The facial depth (mm)
Bjork sum	(N-S-Ar) The sella angle between the anterior and posterior basal planes
	(S-Ar-Go) The articular angle between the posterior basal plane and the ramus
	(Ar-Go-Me) The angular angle between the ramus and the mandibular plane
	The sum of Bjork is the sum of the above angles according to Bjork

^*∗*^The definitions were based on A. Jacobson and R. L. Jacobson [23] and Riolo et al. [24].

**Table 2:** Assessment of the intraobserver reliability and error of the method.

Variable	First measure		Second measure		ICC	Means difference	*p* value^*∗*^
	Mean	SD	Mean	SD			
MdV (mm^3^)	55446	11018	55315	11041	0.999	130.40	0.114
MxV (mm^3^)	63216	7323	63253	7357	0.998	−36.57	0.443
SNA (°)	81.6	3.0	81.5	3.0	0.999	0.02	0.678
SNB (°)	83.2	3.4	83.3	3.4	0.999	−0.07	0.089
ANB (°)	−1.6	4.1	−1.5	4.0	0.999	−0.1	0.811
Y-axis (°)	63.9	3.2	63.8	3.3	0.999	0.05	0.343
MM (°)	23.6	5.6	23.5	5.5	0.999	0.5	0.381
Md.SN (°)	31.8	6.9	31.9	6.9	0.999	−0.02	0.672
Mx.SN (°)	8.31	2.7	8.3	2.8	0.998	0.01	0.782
Bjork sum (°)	391.8	6.9	392.0	6.9	0.998	−0.15	0.613
N-Me (mm)	112.25	5.9	112.2	5.9	0.999	0.06	0.297
S-Go (mm)	74.3	8.7	74.4	8.6	0.999	0.07	0.191
N-Go (mm)	107.5	6.8	107.7	6.7	0.997	0.07	0.089

SD: standard deviation, ICC: intraclass correlation coefficients; MdV: mandibular volume, and MxV: maxillary volume ^*∗*^Comparison between repeated measurements using the paired *t*-test.

**Table 3:** Descriptive statistics of the maxillary volume, mandibular volume, the ratio between the two volumes (MxV/MdV), and the *p* value of significance testing.

Variables	CIII hypodivergent	CIII normodivergent	CIII hyperdivergent	CI normodivergent	*p* value
	Mean ± SD	Mean ± SD	Mean ± SD	Mean ± SD	
MdV	56715 ± 11847	53634 ± 10932	47124 ± 8463	49056 ± 7580	0.017^†^
MxV	66320 ± 12149	64222 ± 12342	56821 ± 10824	62092 ± 10436	0.081^††^
MxV/MdV	1.19 ± 0.20	1.21 ± 0.18	1.21 ± 0.16	1.28 ± 0.20	0.432^††^

Values are given in mm^3^. ^†^Kruskal–Wallis test was conducted. ^††^One-way ANOVA test was conducted.

**Table 4:** The results of significance tests of the mandibular volume between the different studied groups^†^.

Post hoc comparisons		Test value	Corrected *p* value
CIII hypodivergent	CIII normodivergent	13.838	0.441
	CIII hyperdivergent	21.636	0.039^*∗*^
	CI normodivergent	−2.770	1.000

CIII normodivergent	CIII hyperdivergent	18.866	0.067
	CI normodivergent	11.068	0.745

CI normodivergent	CIII hyperdivergent	7.798	1.000

^†^Dann test with Bonferroni's Correction. ^*∗*^Significant at *p* < 0.05.

**Table 5:** Values of the correlation coefficients between 2D variables and the volumetric measurements in CIII hypodivergent and CIII normodivergent patients.

Group	Secondary variable	MdV		MxV	
		*r*	*p* value	*r*	*p* value
CIII hypodivergent	SNA	0.192	0.404	0.425	0.055
	SNB	0.492^*∗*^	0.023	0.455^*∗*^	0.038
	ANB	−0.010	0.967	0.102	0.660
	Y_Axis	−0.301	0.184	−0.500^*∗*^	0.021
	MM	0.144	0.533	0.284	0.211
	MD.SN	−0.555^*∗∗*^	0.009	−0.422	0.057
	MX.SN	−0.582^*∗∗*^	0.006	−0.741^*∗∗*^	0.000
	BJORK	−0.561^*∗∗*^	0.008	−0.504^*∗*^	0.020
	N_Me	0.610^*∗∗*^	0.003	0.411	0.064
	S_GO	0.485^*∗*^	0.026	0.306	0.177
	N_GO	0.566^*∗∗*^	0.007	0.411	0.064

CIII normodivergent	SNA	0.053	0.804	−0.158	0.461
	SNB	0.073	0.736	−0.233	0.272
	ANB	−0.003	0.989	0.013	0.952
	Y_Axis	0.073	0.736	0.104	0.630
	MM	0.401	0.052	0.280	0.186
	MD.SN	−0.045	0.835	−0.094	0.663
	MX.SN	−0.308	0.143	−0.256	0.228
	BJORK	−0.010	0.961	−0.065	0.763
	N_Me	0.782^*∗∗*^	0.000	0.778^*∗∗*^	0.000
	S_GO	0.739^*∗∗*^	0.000	0.763^*∗∗*^	0.000
	N_GO	0.785^*∗∗*^	0.000	0.898^*∗∗*^	0.000

Note: (+) positive correlation; (−) negative correlation; ^*∗*^*r* = 0.4–0.59: moderate correlation; ^*∗∗*^*r* = 0.6–0.79: strong correlation.

**Table 6:** Values of correlation coefficients between 2D variables and the volumetric measurements in CIII hyperdivergent and CI normodivergent patients.

Group	Secondary variable	MdV		MxV	
		*r*	*p* value	*r*	*p* value
CIII hyperdivergent	SNA	−0.202	0.436	0.121	0.644
	SNB	0.198	0.447	0.383	0.129
	ANB	−0.202	0.436	0.391	0.121
	Y_Axis	−0.202	0.436	0.190	0.466
	MM	−0.202	0.436	0.338	0.185
	MD.SN	0.239	0.355	0.241	0.352
	MX.SN	−0.289	0.261	−0.203	0.434
	BJORK	0.249	0.335	0.258	0.318
	N_Me	0.586^*∗*^	0.013	0.262	0.309
	S_GO	0.647^*∗∗*^	0.005	0.384	0.128
	N_GO	0.707^*∗∗*^	0.002	0.434	0.082

CI normodivergent	SNA	0.194	0.386	0.313	0.157
	SNB	0.141	0.531	0.295	0.183
	ANB	−0.057	0.800	−0.024	0.916
	Y_Axis	0.137	0.544	−0.299	0.177
	MM	−0.362	0.098	−0.334	0.129
	MD.SN	−0.479^*∗*^	0.024	−0.119	0.599
	MX.SN	0.090	0.691	0.317	0.150
	BJORK	−0.474^*∗*^	0.026	−0.116	0.608
	N_Me	0.386	0.076	0.148	0.512
	S_GO	0.614^*∗∗*^	0.002	0.138	0.542
	N_GO	0.543^*∗*^	0.009	0.142	0.527

Note: (+) positive correlation; (−) negative correlation; ^*∗*^*r* = 0.4–0.59: moderate correlation; ^*∗∗*^*r* = 0.6–0.79: strong correlation.

## Data Availability

All datasets and spreadsheets are available upon reasonable request to the corresponding author.

## Additional Points

The protocol was not published prior to the trial commencement.
